# Nonspecific Decline in an Elderly Female Revealing a Fatal Infective Endocarditis

**DOI:** 10.7759/cureus.111827

**Published:** 2026-06-30

**Authors:** Kawser Ahmed, Halima Akther, Md Rasel Al Mamun, Moutusi Mehjabin Jyoti, Briste Munda

**Affiliations:** 1 Acute Medicine, Ipswich Hospital, Ipswich, GBR; 2 Acute Medicine, Victoria Hospital, Kirkcaldy, GBR; 3 Accident and Emergency, KPJ Specialized Hospital, Dhaka, BGD; 4 Medicine, Shahabuddin Medical College and Hospital, Dhaka, BGD; 5 Accident and Emergency, BRB Hospitals Limited, Dhaka, BGD

**Keywords:** aortic valve vegetation, cardiac embolic stroke, coagulase-negative staphylococcus epidermidis, infective endocarditis, pulmonary embolism (pe)

## Abstract

We are presenting a case of an elderly female in her 70s who was admitted with extreme fatigue, acute confusion, and functional decline following a respiratory tract infection on the background of multiple complex comorbidities. Initial investigations revealed a chest infection. Therefore, blood culture was requested in view of her advanced age and initial high urea level, which came back positive for *Staphylococcus epidermidis* and the lab report initially considered it a contaminant sample. Further blood cultures were negative. However, she was deteriorating quickly despite proper sepsis cover, which warranted extensive investigations including transesophageal echocardiography (TOE), which revealed a native aortic valve vegetation with peri valvular involvement, confirming native valve infective endocarditis (IE). She was immediately transferred under multidisciplinary team (MDT); however, she failed to improve and died in the hospice a couple days after discharge. This case highlights the diagnostic challenge of IE, especially among frail elderly people who mostly presents with nonspecific symptoms, causing delayed diagnosis and fatal outcome.

## Introduction

Infective endocarditis (IE) is one of the most lethal infections affecting the endocardium and heart valves. It affects multiple organ systems and has a complex clinical presentation. Despite aggressive medical and surgical interventions, the morbidity and mortality are very high. The recent study shows that the mortality has increased significantly within one year of diagnosis, often beyond 30% [1]. There are multiple cardiac (previous IE, valvular heart disease, prosthetic heart valve, central venous or arterial catheter, transvenous cardiac implantable electronic device, and congenital heart disease) and non-cardiac (central venous catheter, people who inject drugs, immunosuppression, recent dental or surgical procedures, recent hospitalization, and hemodialysis) risk factors to develop IE, although the prior IE is the most common risk factor. However, advanced age is a well-established risk factor for infection due to age-related immune system decline and multiple comorbidities. Respiratory infections and bacteremia leading to IE are the most common among elderly people [2,3]. The diagnosis of IE is challenging due to its variable and wide-range clinical features. Not only can it present as an acute aggressive infection, but it also frequently manifests as chronic vague subacute infections, and the latter is more challenging. A systematic review by Alhuarrat et al. emphasized the nonbacterial thrombotic (marantic) endocarditis as an important differential diagnosis of IE [4]. Blood culture, Duke-Li criteria, and echocardiography are the key investigations to diagnose IE. Moreover, the imaging investigations, such as computed tomography (CT) angiogram, cardiac magnetic resonance (CMR), and whole body single-photon emission computed tomography (SPECT)/positron emission tomography/computed tomography (PET-CT), are recommended to rule in the minor diagnostic criteria in case of possible infection endocarditis to yield the correct diagnosis. The main complications of IE are heart failure, uncontrolled infection, and embolism. The treatment options include conservative, medical and surgical. However, the treatment modality will be decided by the endocarditis team [3,5].

## Case presentation

A female in her 70s was admitted to the hospital with progressive fatigue, worsening confusion, reduced oral intake, and functional decline for several weeks. She was initially managed in the community by the general practitioner (GP) and was treated for respiratory tract infection based on her background of chronic obstructive lung disease and bronchiectasis. She received oral antibiotics and steroids for seven days in the community.

Her past medical history was significant for chronic obstructive lung disease, bronchiectasis, coeliac disease, lymphoma, and malignant tumour of transverse colon. On presentation, she was ill-looking, frail, and confused. She was coughing during the conversation and examinations. She was afebrile, and her admission vital signs included a temperature of 37.5℃, heart rate of 90 beat per minute, blood pressure of 100/60 mmHg, respiratory rate of 16 breaths per minute, and saturation of 94% on room air. However, respiratory examinations revealed bilateral coarse crackles more marked on left side, with reduced breath sounds in the left basal area consistent with pneumonia and underlying chronic lung diseases. Cardiovascular examinations were unremarkable. There were no peripheral stigmata of IE. Blood investigations showed chronic anemia with high inflammatory markers, although liver and renal functions were normal except mildly elevated urea (Table 1).

**Table 1:** Laboratory investigations on admission and during hospital course. CRP: C-reactive protein; Hb: hemoglobin; WBC: white blood cells; GFR: glomerular filtration rate.

Parameter	Reference range	Initial value	Subsequent value
CRP	<5 mg/L	114 mg/L	96-104 mg/L
Hb	120–160 g/L	101 g/L	101 g/L (stable)
WBC	4.0–11.0 × 10⁹/L	5.96 × 10⁹/L	5.96-20 × 10⁹/L
Platelets	150–400 × 10⁹/L	145 × 10⁹/L	145-420 × 10⁹/L
Sodium	135–145 mmol/L	129 mmol/L	128 mmol/L
Creatinine	45–90 µmol/L	51 µmol/L	58 µmol/L
Estimated GFR	>90 mL/min	>90	>90
Calcium	2.2–2.6 mmol/L	–	2.15 mmol/L
Folate	>3 ng/mL	–	2.4 (low)

Chest X-ray revealed left-sided consolidation and parapneumonic effusion (Figure 1).

**Figure 1:**
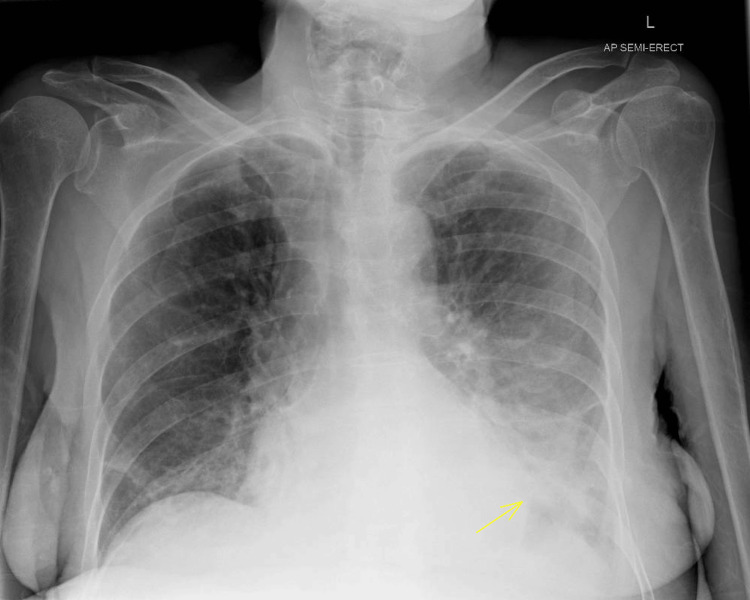
Chest X-ray showing airspace shadowing (yellow arrow) within the left mid and lower zones and small volume left pleural effusion.

Bronchoalveolar lavage (BAL) revealed *Pseudomonas* infection, and the blood culture came back positive for *Staphylococcus epidermidis* and was initially thought to be a contaminant sample (Table 2).

**Table 2:** Microbiological investigations.

Test	Findings
Bronchoalveolar lavage	*Pseudomonas aeruginosa* isolated
Antibiotic sensitivity	Sensitive: amikacin, high-dose ciprofloxacin
Antibiotic resistance	Resistant: ceftazidime, piperacillin/tazobactam
Initial blood cultures	Gram-positive cocci in clusters
Final interpretation	Consistent with *Staphylococcus epidermidis* (likely contaminant)

Despite continued antibiotic, she deteriorated further, both clinically and biochemically. She also developed ascites and splenomegaly (Figures 2, 3).

**Figure 2:**
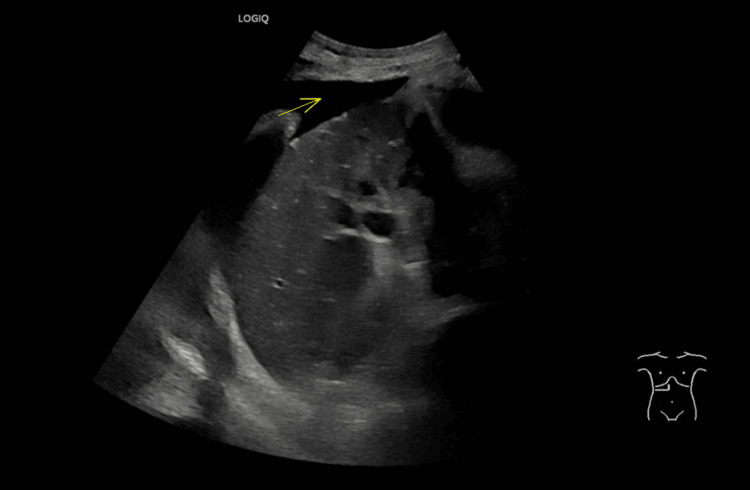
US of liver showing moderate ascites. The yellow arrow shows fluid in the Morison's pouch, consisting with moderate ascites. US: ultrasound.

**Figure 3:**
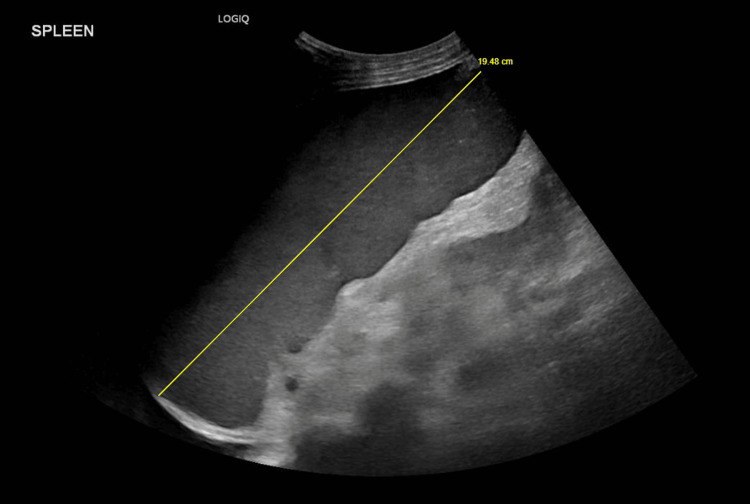
US of spleen showing marked splenomegaly. Yellow line shows the sonographic measurement of spleen: 19.48 cm, consisting with splenomegaly. US: ultrasound.

She developed a bunch of neurological symptoms such as right-sided weakness, facial droop, and a reduction in Glasgow Coma Scale (GCS) from 15 to 7 over the period of time. Hence, CT head had performed, which showed an old infarct (Figure 4). However, she was clinically diagnosed with an ischemic stroke based on her symptoms.

**Figure 4:**
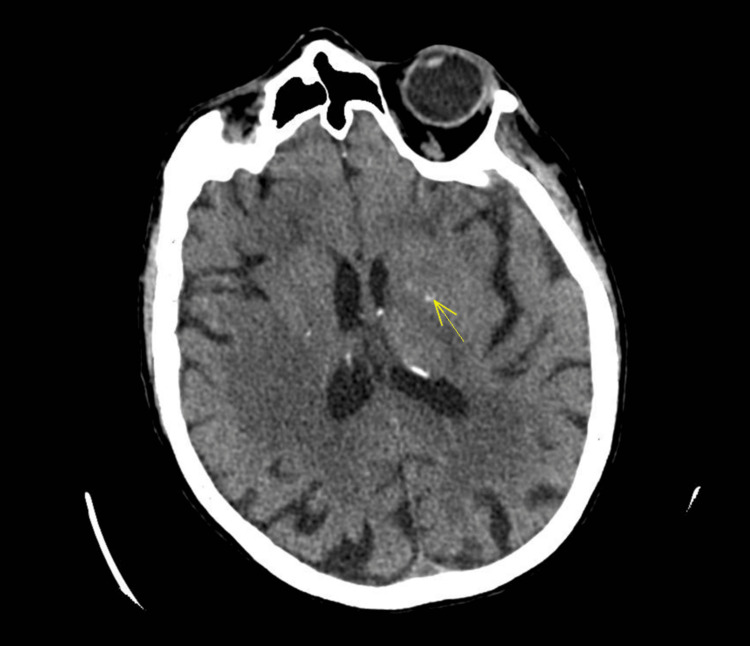
CT scan of the head showing low-density areas, which were thought to be non-recent ischemic changes. The yellow arrow shows tiny hyperdense shadow, consisting age-related changes. The radiologist commented that "please note that CT cannot exclude acute infarction." CT: computed tomography.

Repeat blood cultures came back as negative. However, other investigations revealed embolic phenomenon of IE such as possible embolic pulmonary embolism though she did not have right side IE, possible embolic stroke, splenomegaly, and so on. Although urgent transthoracic echocardiography (TTE) did not show any vegetation or signs of IE, transesophageal echocardiography (TOE) revealed native aortic valve vegetation with perivalvular involvement, confirming the diagnosis of IE, as she met both major diagnostic criteria (blood culture plus imaging). She was managed by IE multidisciplinary team with best evidence-based treatment; however, she failed to improve with a long course of treatment and was finally discharged into the care of a hospice with end-of-life care pathway after 45 days as an inpatient treatment. She unfortunately died a couple of days after discharge from the hospital under the community palliative care team (Figures 5-8).

**Figure 5:**
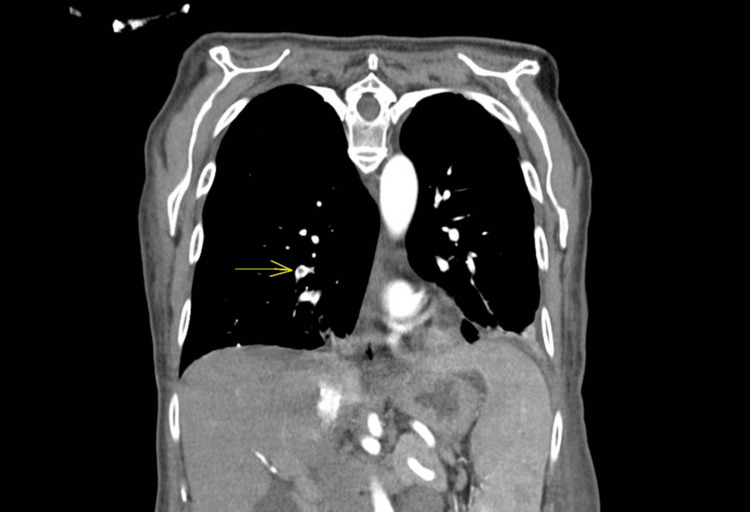
CTPA noted a small PE in the right lower lobe pulmonary arteries branch (yellow arrow). There was also bronchiectasis and bibasal atelectasis with small pleural effusions. CTPA: computed tomography pulmonary angiogram; PE: pulmonary embolism.

**Figure 6:**
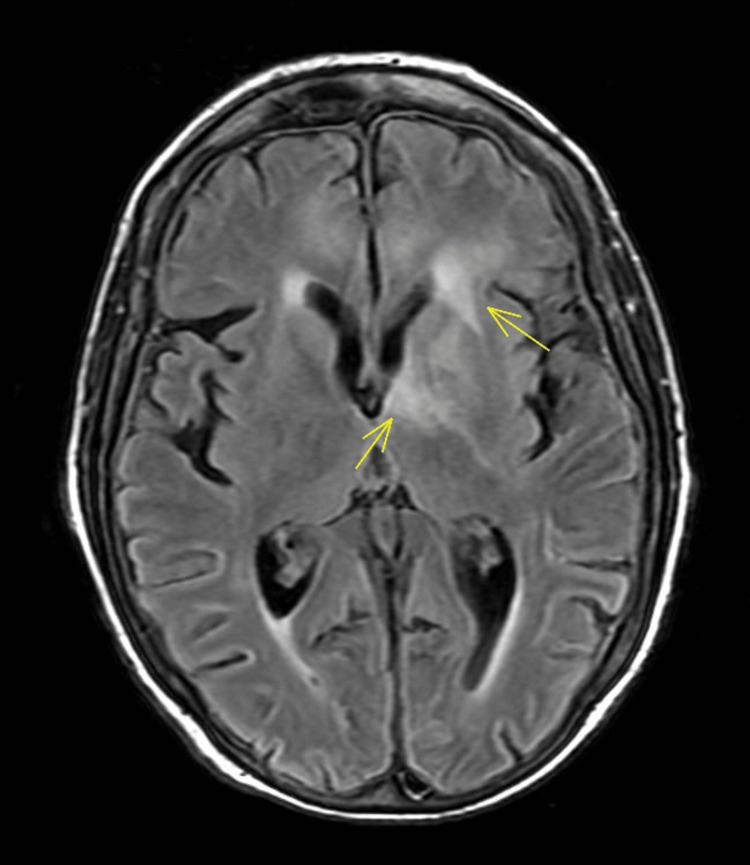
MRI of the head showing hyperintense signal changes in the left basal ganglia and left frontotemporal lobe (yellow arrows), associated with diffuse dural thickening and enhancement. Given the clinical presentation of this patient, it most likely represents an infective etiology causing encephalitis and meningitis. MRI: magnetic resonance imaging.

**Figure 7:**
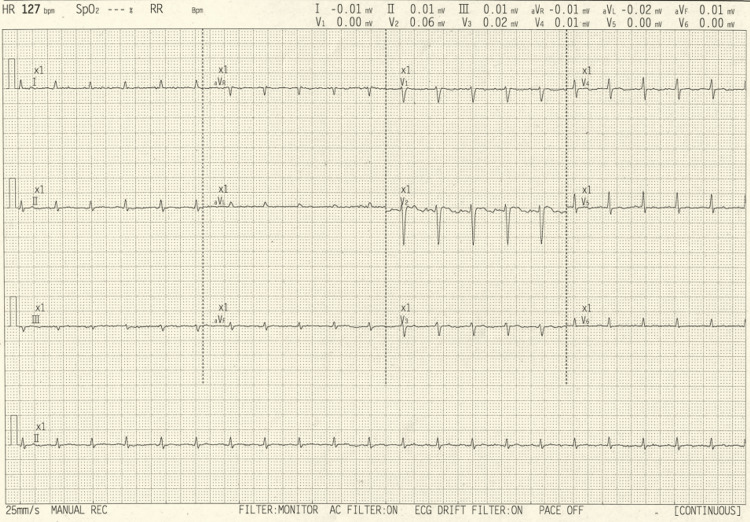
12-Lead ECG showing normal sinus tachycardia and low voltage tracing. ECG: electrocardiogram.

**Figure 8:**
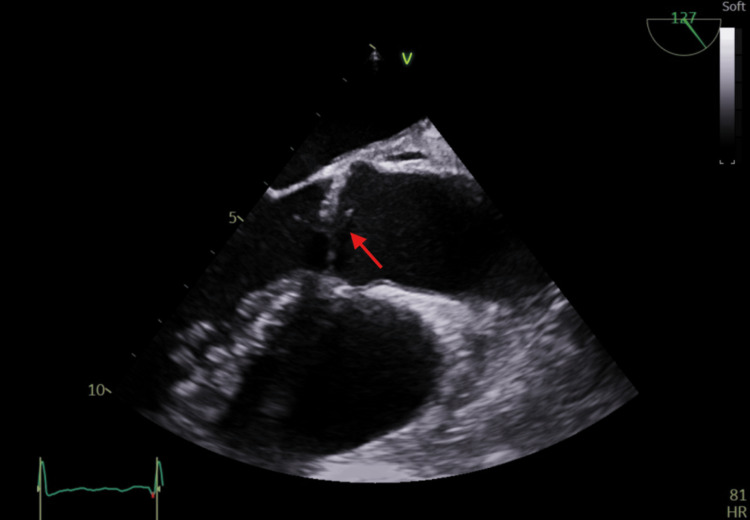
TOE showing 7 mm aortic valve vegetation (red arrow). TOE: transesophageal echocardiography.

## Discussion

This case demonstrates an atypical presentation of IE, pointing several important clinical considerations. Firstly, IE can present with atypical features, especially in elderly or frail people. A comprehensive review by Borkowski et al. uncovered how socioeconomic status operates behind the scenes in the development of IE, along with the established risk factors [6]. Other recent studies have shown that more than one third of the people of Western countries diagnosed with IE are over 70 years [7]. In this case, the predominant features were acute confusion, functional decline, and persistent inflammation/infection, which were initially attributed to a respiratory source, delaying the diagnosis and obscuring the primary pathology. Secondly, coagulase-negative *Staphylococcus epidermidis *bacteremia is a recognized cause of contamination. A recent study by Osaki et al. found that contamination is more common in *Staphylococcus epidermidis* than true bacteremia [8]. Contrarily, *Staphylococcus epidermidis* is also a recognized cause of IE [3]. Therefore, clinical decision should be taken based on individual presentations to avoid any delayed diagnosis. Thirdly, persistent elevation of inflammatory markers without any identifiable etiology should raise suspicion of IE, especially in elderly and frail people [9].

The diagnosis process in this case was unique and makes the case worthy of reporting to make clinicians, such as acute medicine specialist and geriatrician, aware of how an atypical feature may end up leading to a serious diagnosis with a consequent fatal outcome. A decline in functional baseline should be considered a red flag for infection if there are persistent high inflammatory markers despite good antibiotic coverage. Either delirium or new confusion is not only an acute brain failure; sometimes, it is also a cause of persistent heart failure and an unfortunate avoidable loss of precious human life.

From a pathophysiological perspective, *Staphylococcus aureus* is the most common cause of IE. However, *Staphylococcus epidermidis *usually affects the extreme age groups, such as children and elderly people. When the bacteria expose to the endothelium, they cause disruption of endocardial tissue and make it vulnerable to collagen exposure. This subsequently leads to the deposition of fibrin and platelets, ultimately forming the thrombotic vegetation [10].

The treatment of IE is mainly provided by endocarditis multidisciplinary team (MDT). However, it is not always possible in real life to involve an MDT from the beginning of the treatment due to multiple limitations such as delayed diagnosis, limited resources, and lack of interdisciplinary collaboration. According to the 2023 European Society of Cardiology (ESC) Guideline, there are four main prognostic factors at admission for people with endocarditis: (1) patient characteristics (older age, prosthetic heart valve, hemodialysis, and frailty), (2) clinical complications of IE (heart failure, septic shock, and cerebral complications), (3) microbiological features (*Staphylococcus aureus*, fungus, persistent bacteremia, and non-HACEK Gram-negative bacilli), and (4) echocardiographic findings (left-sided IE, periannular involvement, and vegetation size >10 mm). Our patient had many of these, including older age, frailty, cerebral complications, septic shock, *Staphylococcus* species, and left-sided IE, touching all four categories of prognostic factors [3].

There is no consensus guideline or evidence-based input regarding outpatient follow-up of IE after treatment. However, follow-up is usually arranged on individual circumstances to monitor relapse or deterioration in valve function [11]. Unfortunately, our patient died after discharge. Hence, no follow-up was required. Although the overall mortality of IE has been declining from 2007 and is now 7-8%, it remains significantly high in elderly people [12].

## Conclusions

IE is a true diagnostic challenge in atypical presentation, particularly in elderly people. Therefore, clinicians taking care of geriatric groups should be more vigilant in persistent unwell patient and try to find out any deep-seated infections, such as IE, from the beginning of any unsettling infection. Rather assuming contaminated lab results, clinicians should maintain a high index of suspicion in *Staphylococcus epidermidis* bacteremia. Prompt referral and early MDT involvement are crucial to save human lives. Early echo is vital. Hence, even acute medicine specialist or geriatrician should escalate it early to the cardiac imaging specialist for urgent transesophageal echo if suspicion is high. Otherwise, we will lose the valuable life in our daily practice.
